# Medical Experiences: The Doctor

**Published:** 1907-08

**Authors:** 


### MEDICAL EXPERIENCES: THE DOCTOR.

Theresa Bannay says: The science and art of medicine seem to answer more than any other calling the various demands of the intellect for development. All types of physicians are possible. The requirements for the ideal are high. Without being perfect, he must be normal, of good education and decent menners. His common sense and judgment should be sufficient to float him in the tide of knowledge surging upon his professional infancy, to steer him safely between the shores of skepticism and abstruse science, between the nonessential and the necessary. He must not disdain theory, which leads to discovery and progress, nor must he follow its flight, lest the proved ground slip from under his feet. He must be a scientist but cease not to be a practitioner, lest the scientific doubt stay the needful act. His powers of observation need constant exercise, yet if he sees too clearly, his usefulness becomes embarrassment when his patient will not see, or the jury flouts his views. He must be vigorous and decisive, yet kind and gentle, honest and expedient. His morals would seem to be his own concern when they did not affect his professional skill; yet, though a reprobate may be loved with that love akin to pity, it is unstable when respect is lacking. A doctor who is a considerate elder brother to the wife and children and servants within his own home, can be trusted in the homes of others. Such a man is true to the trust of his profession, cherishing it above all personal advantage or gain, keeping it unsullied from the breath of self aggrandizement, unpolluted by commercial measures. With him the honor of his colleagues is safe, their patients secure from defection to his seductive sympathy. To his own patients, weak and miserable, he is strength and comfort, without familiarity or mawkish sentimentality. A father confessor in their shame, a wise counselor in their faltering, a quick actor in their need, a sympathetic friend in their sorrow, a merciful judge in their punishment. This he may be, and is, within the nature of man. To be each of these at all times, in all moods, in every case, is superhuman.—N. Y. Med. Journal, July 6th,

